# Advancing Empirics and Theory for a Deeper Political Economy Analysis: Comment on "Health Coverage and Financial Protection in Uganda: A Political Economy Perspective"

**DOI:** 10.34172/ijhpm.2023.7537

**Published:** 2023-09-10

**Authors:** Ashley M. Fox

**Affiliations:** Rockefeller College of Public Affairs and Policy, University at Albany, SUNY, Albany, NY, USA

**Keywords:** Political Economy, Universal Health Coverage, Low- and Middle-Income Countries

## Abstract

At its core, political economy analysis involves examination of the relationship between the state and the market. A number of country case studies have emerged in recent years that aim to identify political economy factors facilitating or impeding health sector reforms towards universal coverage. In this commentary, we expand Nannini and colleagues’ analysis to elaborate on how political economy analyses can better inform policy design towards more successful reforms in low- and middle-income countries (LMICs) by drawing more heavily on improved research design and theory. We suggest three ways that political economy studies could make deeper claims by historicizing analyses, going comparative and/or by grounding findings more deeply in theory.

 When and why do countries adopt health financing reforms that ensure that access to health services is based on need rather than ability to pay? Nannini et al^1^ undertake a study of the political economy of universal health coverage (UHC) reform in Uganda and find that the current political situation is not yet conducive for implementing a UHC system with widespread financial protection. They identify several challenges specific to the case of Uganda, including the following: (1) Unions oppose payroll deductions from workers’ pay as a financing mechanism; (2) The “pre-payment” mechanism requiring contributions from members contradicts the notion that care should be “free” leading to reduced popularity of the proposed reform; (3) A growing private health sector fears competition between social health insurance and commercial schemes; and (4) Low confidence in government capacity. These are important insights and each are quite consistent with both theoretical expectation and other case studies of health reforms towards UHC in low- and middle-income countries (LMICs).^2^

 A number of single case-studies of applied political economy analyses such as Nannini and colleagues’ have arisen in recent years in global health that are aimed either at some exercise of prediction (ie, is a country likely to adopt a given reform?) or explanation (ie, why a country did or did not adopt a particular reform at a specific moment?).^3-6^ A recent systematic review by Rizvi et al^7^ identified at least 55 papers on the political economy of health reform, most of which were individual case studies. These studies have offered useful insights about the particulars of specific cases of reform. However, this commentary argues that the time has come to move beyond description to advance a more rigorous application of empirics and theory in political economy studies of health reform in LMICs to improve our ability to generalize beyond specific cases and make broader claims and applied recommendations.

 Three specific ways that political economy studies of health reform in LMICs could make deeper claims include the following:

1. Historicize analyses
2. Go comparative
3. Test competing theories and/or ground findings more deeply in theory

## Historicize Analyzes

 Historical institutionalism is an approach to political economy analysis that emphasizes how timing, sequences and path dependence affect the development of institutions over the long term and shape future policy paths.^8^ In other words, by setting countries down a particular path, past policies are one of the strongest predictors of future policies. For instance, in a US context, policies adopted in the 1950s that encouraged the proliferation of private, employer-sponsored health insurance has created expectations about how healthcare should be provided and a powerful industry supporting its continuation as a private good. These types of self-reinforcing trends are known as “policy feedback” effects.^9^ Research on policy feedback finds that not only does politics shape policy choices, but also that past policy choices shape future politics. This occurs because new policies create constituencies who benefit from a particular policy and whose political consciousness is shaped by their experiences with existing policies and programs. For instance, social security policies that are targeted at older adults bestow particularistic benefits upon this group, which elevates their consciousness and motivation as a powerful political constituency.^10^ Likewise, by examining the evolution of health sector reforms over nearly 60 years, Harris and Libardi Maia^11^ find that path-dependent processes that entrenched the private sector in Brazil have pushed Brazil and Thailand in divergent reform directions despite a similar effort/interest in building universal coverage. These are the types of insights that can be obtained by historicizing analyses.

 By contrast, recent studies claiming to apply political economy analysis tend to focus largely on the present moment. Nannini et al^1^ are no exception, focusing largely on the last two decades. Based on this analysis, they conclude that “the current political situation is not yet conducive for implementing a UHC system with widespread financial protection: dominant interests and ideologies do not create a net incentive to implement a comprehensive scheme for this purpose.” However, an analysis based solely on the present moment, or recent past, begs the question of how this moment compares to those that have come before. Is the present moment especially unconducive when compared against prior moments? Historizing accounts can provide insights into how and why present institutions have evolved in the way they have – and how these institutional arrangements might influence present and future reform prospects. Additionally, political economy theory and empirical analysis suggests that the conditions conducive to reform often emerge and converge rapidly,^12^ rendering the present moment potentially a poor predictor of future policy openness without additional context over a longer time horizon.

 Though there is no recipe for how long is appropriate to look back, by employing different “periodization” strategies to test the effects of institutional changes or exogenous shocks,^13^ studies employing political economy analysis can better identify relevant time frames for analysis. For example, by placing their analysis in the historical trajectory of Zimbabwe’s health reforms since the country’s independence in the 1980s, Mhazo and Mapongo^6^ find that the political opportunity structures for reform in Zimbabwe have changed in recent decades, with the window for reform shrinking. While Mhazo and Mapongo^6^ come to a similar conclusion as Nannini et al,^1^ their longer time horizon offers more confidence that this moment is less conducive than those that have come before. Similarly, Nannini et al^1^ could have looked to the nature of the National Resistance Movement regime, or previous regimes, in Uganda and what they have meant for Uganda’s health sector over time.

 From a practical perspective, how might historicizing analyses matter? By historicizing key policies, researchers can better identify available pathways for reform and policymakers can avoid policy choices that are likely to create institutional arrangements that will be difficult to diverge from in the future. For instance, Nannini and colleagues’^1^ finding that unions oppose payroll deduction from workers’ pay is understandable when cast in light of existing institutional arrangements that have evolved to benefit particular classes of workers – ie, those in the formal sector with union protections. Contestations over fringe benefits are quite common under corporatist arrangements that require tripartite negotiations among labor, state and industry.^14^ However, in a context of low formal sector employment, rather than this institutional arrangement presenting an opportunity for more comprehensive reform, labor unions representing formal-sector workers may also fear how new national programs might steal funds from or otherwise dilute their own existing state (social security) programs for formal sector workers.^2^

## Go Comparative

 A second approach to advancing potential lesson drawing is to use small-N case comparisons to draw inferences. Small-N comparative case studies can be powerful tools that can simulate larger N quantitative designs by attempting to reduce “endogeneity” through the process of case selection to isolate major explanatory factors. A well-done comparative case study not only makes the case for a particular causal explanation, but also tries to systematically rule out alternative explanations. Comparative case studies thereby aim to move from description to explanation.^15^ However, well-done comparative case studies are rare in global health. This is likely due to multiple reasons including the difficulty of identifying appropriate case comparisons, low incentives for researchers to devote the necessary time and resources to develop deep knowledge of multiple cases, the fact that a lot of research arises from short-term consultancies, and lack of training in these methods, among others.

 For instance, Nannini and colleagues’ findings that unionized workers tend to oppose extending national health insurance via mechanisms that would impose a payroll tax could be examined across a larger set of cases to determine whether this is a more generalized source of resistance to insurance-based reform in LMICs or is more specific to Uganda. In fact, recent studies and reviews of literature have tended to find support for the notion that unions will tend to oppose coverage expansions that are financed through payroll taxes that tend to fall heavier on formal sector workers.^2^ If union opposition is theorized to be a major barrier to reform, cases could be selected to compare countries with different outcomes to examine how opposition from unions was overcome. Or countries could be matched based on their union strength or bargaining mechanisms to assess whether outcomes vary based on this factor. A recent 11-country study of countries at different stages of UHC identified useful lessons for countries moving towards coverage expansions.^16^ Another example of a successful comparative case study that simultaneously integrates history and comparative design includes Wong’s book, ‘*Healthy Democracies,*’ which employs a most similar systems design to identify the role that democratization in Taiwan and South Korea played in changing the incentive structures of vote-seeking policymakers in favor of popular social policies – ie, UHC.^17^ Similarly, in his 2017 book, ‘*Achieving Access,*’ Harris compares efforts to institutionalize universal healthcare and expand access to AIDS drugs in three major industrializing countries: Thailand, Brazil, and South Africa.^18^ He finds that democratization empowered elite professional associations to advocate for universal healthcare and AIDS treatment. Well formulated comparative designs such as this enhance the internal validity and inferences that can be drawn from small-N studies and follow a similar causal logic to larger-N studies.^15^

 By going comparative, researchers can seek to answer such questions as where have UHC reforms been successful, where have they failed, and where have they simply not been tried? Which explanations for low agenda status of UHC recur across different settings? Which factors affecting UHC expansions appear to be more general versus case specific? Where has the private sector been successfully kept at bay? What are the roles of regime type, social movements, professional associations or political leadership in UHC expansions?

## Test Competing Theories and/or Ground Findings More Deeply in Theory

 Research on the political economy of health reform in global health tends to be theory-light. Frameworks are frequently developed and applied in a rote manner with hypotheses never clearly specified. Inductive approaches are utilized to examine emergent factors that mattered in a specific case, but broader theories or expectations are rarely formed that could be extended to other cases. Yet, contributions to broader theory is what separates academic research from policy reports that recount events but whose implications fail to extend beyond the particular case at hand. To enhance the generalizability of findings, political economy analyses of health reform can test competing theories or compare case specific elements against expectations derived from theory. Of note, these ‘empirical tests’ need not be quantitative in nature. Rather, they may involve a variety of qualitative approaches, which can help illuminate causal mechanisms.^13,15^

 Political economy studies of health reform that better incorporate theory and more explicitly test different theoretical mechanisms have identified several explanations for when and why major redistributive programs might emerge (see eg, Hall^19^ for a summary of major explanations). These include interest-based explanations, which suggest that groups or individuals have economic “interests” that undergird their behavior in predictable ways and that individuals/groups will behave in a rational manner to enhance their influence and power.^19^ Interest-based explanations might predict that countries with a strong private sector and powerful public sector unions might oppose payroll based reforms that would work against their immediate economic interests. Institutions-based explanations do not necessarily dispute interest-based accounts, but note that interests are “structured” or channeled in certain predictable ways by the “institutional” arrangements they confront in different national contexts. For instance, institutions-based explanations might look at how democratization changes the relative power of different interest groups and their ability to influence health reform as a number of studies have.^17,18^ Explanations that emphasize the power of ideas, by contrast, put primacy on the ways that ideas shape policy debates independent of the material interest that undergird them. Ideas-based explanations might look to whether there are influential social movements that frame healthcare as a human right or whether public opinion is strongly supportive of or against specific reforms. Likewise, explanations emphasizing a combination of ideology and organizational strength, such as power-resources theory,^14^ have suggested that left parties will tend to champion reforms as a consequence of the linkages between left parties and labor unions though recent studies have recognized this may not work the same way in an LMIC context where party ideology does not neatly fit a left-right continuum.^2,20^

 In the absence of theory testing, studies risk concluding that ‘everything matters’ or ‘it depends,’ which may not be very helpful in advancing policy or practice. In the absence of theory testing, what stable recommendations can we make to policy reformers that their proposed reforms will be likely to succeed?

 An example of the successful application of theory to a single case might be Croke et al,^3^ who showed that popular opinion against moving from a national health service model to a national health insurance model was a major factor undermining health reform towards UHC in Malaysia. Likewise, Harris^2^ finds that left leaders in LMICs will tend to oppose insurance-based models, which are seen as more “neoliberal” than health-service based models. These studies support Nannini and colleagues^1^ finding that insurance-based reforms in Uganda has been undermined by the notion of “pre-payment” via an earmarked payroll tax, which contradicts the government’s commitment to “free” healthcare. According to Nannini et al, moving to an insurance-based model is seen as both compromising the public popularity of UHC and the political calculus associated with its extension. This finding could be understood as an “ideas”-based explanation, grounded in notions of public opinion and policy responsiveness.

 These approaches to deepening political economy analysis of health reform are not mutually exclusive. Comparative studies may look historically to identify relevant cases or examine change over time within cases. Historical institutional and comparative studies should incorporate theory to develop their hypotheses or “priors” about expectations. These approaches can also help identify whether and how theories developed in high-income country contexts are directly applicable to present-day LMIC contexts or if expectations need to be modified/updated.^20^

 In summary, the growing number of quality case studies of the political economy of UHC in LMICs is a welcome development. We applaud the growing recognition that health reform is a political and not merely a technical process. However, we believe the breadth of insights derived from political economy studies of UHC in LMICs could be strengthened through attention to improved methodological and theoretical approaches to political economy analysis.

## Acknowledgements

 I would like to thank Dr. Michael Reich for his helpful feedback and suggestions on the Comment as well as the three anonymous reviewers whose input helped improve the final version.

## Ethical issues

 Not applicable.

## Competing interests

 Author declares that she has no competing interests.
